# Low expression of *ACLY* associates with favorable prognosis in acute myeloid leukemia

**DOI:** 10.1186/s12967-019-1884-5

**Published:** 2019-05-10

**Authors:** Jinghan Wang, Wenle Ye, Xiao Yan, Qi Guo, Qiuling Ma, Fang Lin, Jiansong Huang, Jie Jin

**Affiliations:** 1 Department of Hematology, The First Affiliated Hospital, Zhejiang University College of Medicine, No. 79 Qingchun Road, Hangzhou, 310003 Zhejiang People’s Republic of China; 2 Institute of Hematology, Zhejiang University, Hangzhou, People’s Republic of China; 3 Key Laboratory of Hematologic Malignancies, Diagnosis and Treatment, Hangzhou, Zhejiang People’s Republic of China; 4 Department of Hematology, Qingdao Municipal Hospital, Qingdao, Shandong China; 5 Department of Nephrology, The First Affiliated Hospital, Zhejiang University, Hangzhou, China; 6 Department of Hematology, The Second Affiliated Hospital of Henan University of Traditional Chinese Medicine, Zhengzhou, China

**Keywords:** ATP citrate-lyase, Acute myeloid leukemia, Prognosis

## Abstract

**Background:**

Aberrant metabolism is a hallmark of cancer cells. Recently, ATP citrate-lyase (*ACLY*) expression was demonstrated as an independent predictor of clinical outcome in solid tumors. However, no systematic study was conducted to explore the prognostic impact of *ACLY* on acute myeloid leukemia (AML).

**Methods:**

To assess the prognostic value of *ACLY* transcript, we conducted a study with a discovery and validation design. We measured *ACLY* transcript by real-time quantitative PCR in 274 AML patients, and validated the prognostic value in the two independent cohorts using published data. Meta-analysis of gene-expression profile and inhibition *ACLY* expression in leukemia cell lines were conducted to help us understand the biological insight of low *ACLY* expression.

**Results:**

Low *ACLY* expression is less common amongst AMLs with *DNMT3A* mutations, but coexisted in double allele *CEBPA* mutations. Moreover, low *ACLY* expression is associated with favorable overall survival in AML patients and is involved in multiple pathways. These results are also validated in two independent cohorts of AML patients. Moreover, *ACLY* silencing induces proliferation arrest in THP-1 and MOLM-13 leukemia cell lines.

**Conclusion:**

We found low *ACLY* expression is associated with favorable overall survival in AML patients.

**Electronic supplementary material:**

The online version of this article (10.1186/s12967-019-1884-5) contains supplementary material, which is available to authorized users.

## Background

Acute myeloid leukemia (AML) represents a group of heterogeneous hematopoietic malignant diseases. To date, substantial progress has been made in the understanding of AML pathogenesis with respect to genomic or proteomic abnormalities, but our knowledge about the metabolic behavior of leukemia is far from satisfactory. Recently, we conducted a series of studies on the link between serum metabolites or cytogenetic abnormalities and treatment response in order to investigate novel biomarkers in AML patients [1–4]. Notably, we found that increased levels of fatty acids and TCA intermediates are associated with higher risk of cytogenetic subtypes, which implied a worse clinical outcome [1, 3]. This result reflects the metabolic behavior of leukemia cells that up-regulated fatty acid synthesis, which might fuel membrane biogenesis and thereby affect therapy response [5, 6]. In addition, we observed that enhanced *IDH1* expression is also associated with poor prognoses [4]. In humans, increased activity of the IDH1 enzyme facilitates lipid synthesis [6, 7]. It is worth noting that ATP citrate-lyase (ACLY) is a lipogenesis enzyme, converting citrate to cytosolic acetyl-CoA [7]. Acetyl-CoA is not only involved in fatty acid synthesis, but also in regulating multiple pathways [8, 9]. Moreover, a growing number of studies demonstrated that high *ACLY* expression is an independent predictor for inferior clinical outcome in several types of cancers, including lung, liver, and gastric adenocarcinoma [6, 10–14]. However, to the best of our knowledge, there is not yet a study to exclusively evaluate the clinical significance and biological insights of *ACLY* expression in AML. To address this unresolved issue, we evaluated the prognostic value of *ACLY* expression in our AML patients and validated the result against two published cohorts of AML patients. At the same time, we provide several critical pathways associated with low *ACLY* expression, and demonstrate that inhibition of *ACLY* led to proliferation arrest in THP-1 and MOLM-13 leukemia cell lines. Thus, *ACLY* expression in AML may act as a prognostic predictor and potential therapy target in the near future.

## Materials and methods

### Patients

Clinical data were collected from the medical records of AML patients at the Zhejiang Institute of Hematology (ZIH) in Zhejiang Province, China. Between March 2010 and June 2017, 274 patients with detailed diagnoses and treatment information were enrolled. We excluded patients with acute promyelocytic leukemia in this study. WHO classification, cytogenetic risk classification and treatment protocols were reported previously [2, 4, 15]. Cellular materials were stored at the department of hematology in our hospital. The details can also be seen in Additional file 1. In addition, 165 patients with survival information from published data on Gene Expression Omnibus using the Affymetrix Human Genome U133A Array platform (accession number: GSE1159) and 197 patients from TCGA (https://tcga-data.nci.nih.gov/tcga/) were set as the independent validation cohorts [16].

### Quantitative real-time PCR

Total RNA was isolated according to the manufacturer’s instructions using RNAiso plus (Takara, Japan). Reverse-transcription polymerase chain reaction (qRT-PCR) was carried out using Invitrogen RT kit according to the manufacturer’s instructions (Invitrogen, USA). *ACLY* mRNA expression was conducted by qRT-PCR with co-amplification of the reference gene *ABL1*. The relative expression levels of *ACLY* were measured by 2^−ΔΔCt^ [4]. The primers sequences of *ACLY* and *ABL1* are described in Additional file 1: Table S1.

### Cytogenetic and gene mutation analysis

We isolated patients’ bone marrow (BM) samples at primary diagnosis through Ficoll gradient centrifugation. *FLT3*-ITD, *NPM1* and *CEBPA* gene mutations were analyzed as previously described [1]. Detailed methods are available in Additional file 1.

### Cell culture and knockdown of *ACLY*

The AML cell line THP-1, Kasumi-1, NB4 and HL-60 were purchased from the Shanghai Cell Culture Institute (Shanghai, China). MV4-11, MOLM-13, OCI-AML3 cell lines were provided generously by Professor Ravi Bhatia (City of Hope National Medical Center, Duarte, CA). Human *ACLY* shRNA lentivirus plasmid was obtained from Genecopoeia (USA). The targeting sequences of each shRNA are shown in Additional file 1: Table S2. The Colorimetric CellTiter 96 Aqueous One Solution Cell Proliferation Assay (MTS assay, Promega, Madison, WI, USA) was used to measure cell proliferation. The methods for cell cultures, *ACLY* knockdown and detection of cell viability are detailed in Additional file 1.

### Definition of clinical endpoints and statistical analysis

The main objective of this study was to evaluate the prognostic impact of *ACLY* expression on the overall survival (OS) of AML patients. *ACLY* expression level relative to that of the reference gene *ABL1* was used for analysis in this study. To determine the optimal cutoff value of *ACLY* expression in predicting OS, we carried out a log-rank test analysis using the Cutoff Finder software package [17]. The prognostic value of *ACLY* expression obtained from our patients was also validated against the independent data obtained from GSE1159 and TCGA, using with the same method to estimate the optimal cutoff value of *ACLY* expression. Patient characteristics were summarized using descriptive statistics, which included frequency counts, median and range. The relationship between *ACLY* expression and patient characteristics was evaluated by a nonparametric test and Fisher’s exact test. The primary endpoint of the study was OS. OS was measured as time from disease diagnosis to death from any cause, or censoring for patients alive at their last known date of contact. The Kaplan–Meier (KM) method for univariate analysis and the Cox proportional hazard regression model for multivariate analysis were used to determine the independent prognostic value of *ACLY* expression levels. The proportional hazard assumption and linear relationship were checked when constructing the Cox regression model. Differently expressed genes associated with low *ACLY* expression were determined through meta-analysis using the “MetaDE” package [18]. KEGG pathways associated with low *ACLY* expression were analyzed using the processed data from the NCBI gene Expression Omnibus GSE1159 and TCGA. All statistical analyses were conducted with R statistic package, version 3.1.3 (http://www.r-project.org). P < 0.05 demonstrated statistical difference.

## Results

### Patient characteristics

In this study, *ACLY* expressions were analyzed in bone marrow samples from 274 adult patients with newly diagnosed AML. The relative transcript expression levels of *ACLY*/*ABL1* ranged from 0.012 to 1.303, with a non-normal distribution in this cohort (Additional file 1: Figure S1A, B). 172 of 274 (63%) AML patients were male. At diagnosis, patients with low *ALCY* expression had significantly lower WBC counts, but high levels of hemoglobin (Table 1). Interestingly, low *ACLY* expression were almost always accompanied by *CEBPA* double allele mutations, but mutually exclusive with *DNMT3A* mutations (P = 0.005). There was no significant correlation between *ACLY* expression and other clinical parameters including age, sex, platelet counts, blast percentage, cytogenetic risks, genes of *NPM1*, *FLT3*-ITD mutations and treatment protocols.

**Table 1:** Clinical characteristics of patients with low *ACLY* expression

Variable	Low expression	High expression	P value
Number	58	216	
*ACLY*, median (range)	0.14 [0.12, 0.16]	0.33 [0.25, 0.40]	< 0.001
Sex, female, n (%)	38 (65.5)	134 (62.0)	0.650
Age, median (range), years	45.50 [31.50, 62.75]	49.00 [35.00, 60.00]	0.468
WBC, median (IQR), ×10^9^/L	13.55 [4.03, 34.00]	25.20 [5.65, 87.00]	0.010
HB, median (IQR), g/L	87.00 [73.00, 102.00]	78.80 [65.00, 97.00]	0.044
PLT, median (IQR), ×10^9^/L	42.50 [22.00, 70.75]	42.00 [21.00, 77.00]	0.980
BM blast, median (IQR), %	65.75 [49.62, 81.25]	70.14 [52.34, 84.25]	0.541
FAB classification, n (%)			0.166
M0	3 (5.2)	21 (9.7)	
M1	7 (12.1)	20 (9.3)	
M2	29 (50.0)	86 (39.8)	
M4	6 (10.3)	20 (9.3)	
M5	11 (19.0)	62 (28.7)	
M6	2 (3.4)	1 (0.5)	
AML	0 (0.0)	6 (2.8)	
Cytogenetic risks, n (%)			0.110
Favorable	9 (15.5)	15 (6.9)	
Intermediate	42 (72.4)	177 (81.9)	
Poor	7 (12.1)	24 (11.1)	
Gene mutations, n (%)			
*FLT3*-ITD	6 (11.5)	30 (14.6)	0.660
*CEBPA*^DM^	10 (20.0)	10 (5.0)	0.002
*NPM1*	12 (22.2)	56 (26.7)	0.602
*DNMT3A*	1 (1.8)	23 (10.8)	0.035
Treatment, n (%)^a^			0.982
DA	22 (37.9)	78 (36.1)	
HAA	17 (29.3)	63 (29.2)	
IA	19 (32.8)	75 (34.7)	

*WBC* white blood cell, *HB* hemoglobin, *PLT* platelet counts, *BM* bone marrow, *FAB* French–American–British classification systems, *DM* double-allele, *IQR* interquantile

^a^The protocols used for induction therapy in different groups including HAA, homoharringtonine-based treatment (homoharringtonine 2 mg/m^2^/day for 3 days, cytarabine 75 mg/m^2^ twice daily for 7 days, aclarubicin 12 mg/m^2^ daily for 7 days) regiment; DA, daunorubicin 45 mg/m^2^ daily for 3 days and cytarabine 100 mg/m^2^ daily for 7 days; IA, idarubicin 6–8 mg/m^2^ daily for 7 days and aclarubicin 20 mg/m^2^ daily for 5 days

### Prognostic significance of *ACLY* expression in Chinese AML patients

**Fig. 1:**
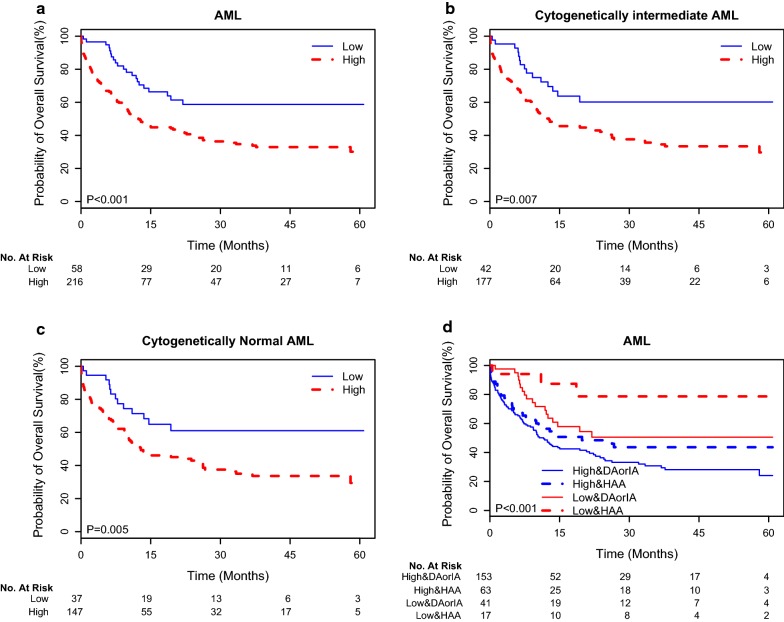
Survival analyses of patients with AML. Kaplan–Meier estimates of OS by high and low *ACLY* expression for AML patients (**a**), cytogenetically intermediate AML (**b**) cytogenetically normal AML (**c**), and stratification of distinct *ACLY* levels and treatment protocols (**d**), respectively. The protocols include homoharringtonine-based regiment (HAA), daunorubicin and cytarabine (DA), idarubicin and aclarubicin (IA)

In the entire cohort of AML, the 3-years OS rate of our patients was 40%. Here, we have taken *ACLY* expression as both a continuous variable and multiple categorical variables (Additional file 1: Figure S2), and found they were statistically associated with poor OS [as a continuous variable, HR (95% CI), 3.54 (1.67, 7.52); P = 0.001]. Using a cutoff value of *ALCY* expression determined using Cutoff Finder, we organized the patients into high and low *ALCY* expressers. Low *ACLY* expressers (n = 58, 21%) had a more favorable OS compared to high expressers (n = 216) (Fig. 1a). Importantly, in the subgroup analyses we found low *ACLY* expressions were associated with favorable OS in patients in both the cytogenetic intermediate risk group and the cytogenetically normal AML group (Fig. 1b, c). In addition, our univariate analysis demonstrated statistically significant, adverse survival effects resulting from older age, increased WBC counts, cytogenetically high risk and *FLT3*-ITD and *DNMT3A* gene mutations. Factors that imparted favorable survival impacts were double allele *CEBPA* mutations and *MPN1* mutations (Table 2). Even if these and induction chemotherapy protocols are taken into account as confounding factors, *ACLY* expression was still effective as an independent prognostic factor in the multivariate analysis [HR (95% CI), 1.805 (1.062, 3.068); P = 0.029; Table 2]. Of note, we found that patients treated with HAA regimens had higher overall survival than those received with IA or DA by high and low *ACLY* expressions (Fig. 1d).

**Table 2:** Univariate and multivariate analysis of AML patients for overall survival

Variables	Univariate analysis		Multivariate analysis	
	P value	HR (95% CI)	P value	HR (95% CI)
ACLY expression (high vs. low)	0.001	2.19 (1.38, 3.475)	0.029	1.805 (1.062, 3.068)
Age	< 0.001	1.007 (1.004, 1.011)	0.009	1.006 (1.001, 1.01)
WBC	< 0.001	1.005 (1.003, 1.007)	< 0.001	1.004 (1.002, 1.006)
Cytogenetic risks				
Intermediate vs. favorable	0.003	3.811 (1.556, 9.337)	0.027	3.748 (1.164, 12.069)
Poor vs. favorable	< 0.001	8.539 (3.247, 22.454)	0.001	7.978 (2.34, 27.197)
Gene mutations (mutation vs. wild-type)				
*FLT3*-ITD	0.022	1.66(1.076, 2.56)	0.007	1.969(1.208,3.21)
*NPM1*	0.051	1.434(0.998,2.061)	0.812	1.05(0.702,1.571)
*CEBPA*^*DM*^	0.022	0.385(0.169,0.874)	0.040	0.409(0.175,0.960)
*DNMT3A*	0.001	2.474(1.483,4.125)	0.022	1.98(1.105,3.546)
Treatment^a^				
HAA vs. DA	0.001	0.503 (0.335, 0.756)	0.007	0.536 (0.341, 0.842)
IA vs. DA	0.003	0.567 (0.391, 0.824)	0.002	0.48 (0.304, 0.756)

Age and WBC are taken as continuous variables

*WBC* white blood cell, *DM* double-allele, *CI* confidence intervals, *HR* hazard ratio

^a^The treatment protocols are available from Table 1

### Low expression of *ACLY* was associated with favorable overall survival in two published datasets

**Fig. 2:**
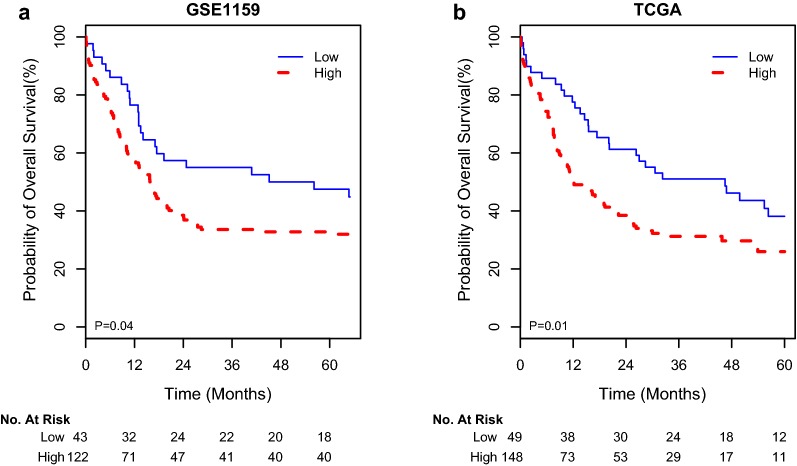
Kaplan–Meier survival curves of patients with high and low *ACLY* expression in two validated cohorts

To further evaluate the prognostic impact of *ACLY* expression, we analyzed the GSE1159 dataset and TCGA dataset. The median relative transcript expression levels of *ACLY/ABL1* were 1.53 and 3.75 in the GSE1159 and TCGA datasets, respectively. We then subdivided patients into high and low subgroups using the same statistical analysis methods used in our cohort. As a result, 43 of 165 (26%) patients in GSE1159 dataset and 49 of 197 (25%) in the TCGA cohort were identified as low *ACLY* expressing. In these two cohorts, patients with low *ACLY* expression had a favorable OS compared to those with high expression levels (Fig. 2). Additionally, low *ACLY* expression in the TCGA dataset was also shown to be negatively associated with WBC count (Additional file 1: Table S3). Finally, the prognostic factor of *ACLY* expression remained significant in the multivariable analysis after adjusting for the most well-established predictors of OS, such as age, WBC count, cytogenetic risks and *FLT3*-ITD, *NPM1* and *DNMT3A* gene mutations in the TCGA cohort (Additional file 1: Table S4).

### Meta-analysis of gene-expression profile in AML patients with low *ACLY* expression

We applied a meta-analysis of mRNA expression to our 22 CN-AML patients with previously published gene expression dataset [15], 165 patients from the GSE1159 dataset and 197 patients from the TCGA dataset profiles to explore the biological insights of low *ACLY* expression. We found 390 genes were down-regulated and 111 genes were upregulated in the low *ACLY* group using the false discovery rate (FDR < 0.05; Additional file 1: Table S5, Figure S3). In the KEGG pathways enrichment analysis, we found low *ACLY* expressers involved in 27 downregulated pathways, including pathways linked to oxidative phosphorylation, proteasomes, Huntington’s disease and the citric acid cycle (Additional file 1: Table S6). In contrast, although none of the upregulated genes were enriched in the KEGG pathways, we found 12 genes (*SPRY2, DNAH3, TRAF5, SFSWAP, ARID5A, ME3, BAZ2A, ARHGEF7, CD99, CSNK1E, GNA15* and *POFUT2*) in patients with pediatric acute myeloid leukemia bearing an inv(16) translocation [19] were upregulated in low *ALCY* expressers.

### Inhibition *ACLY* expression induces growth arrest in AML cell lines

**Fig. 3:**
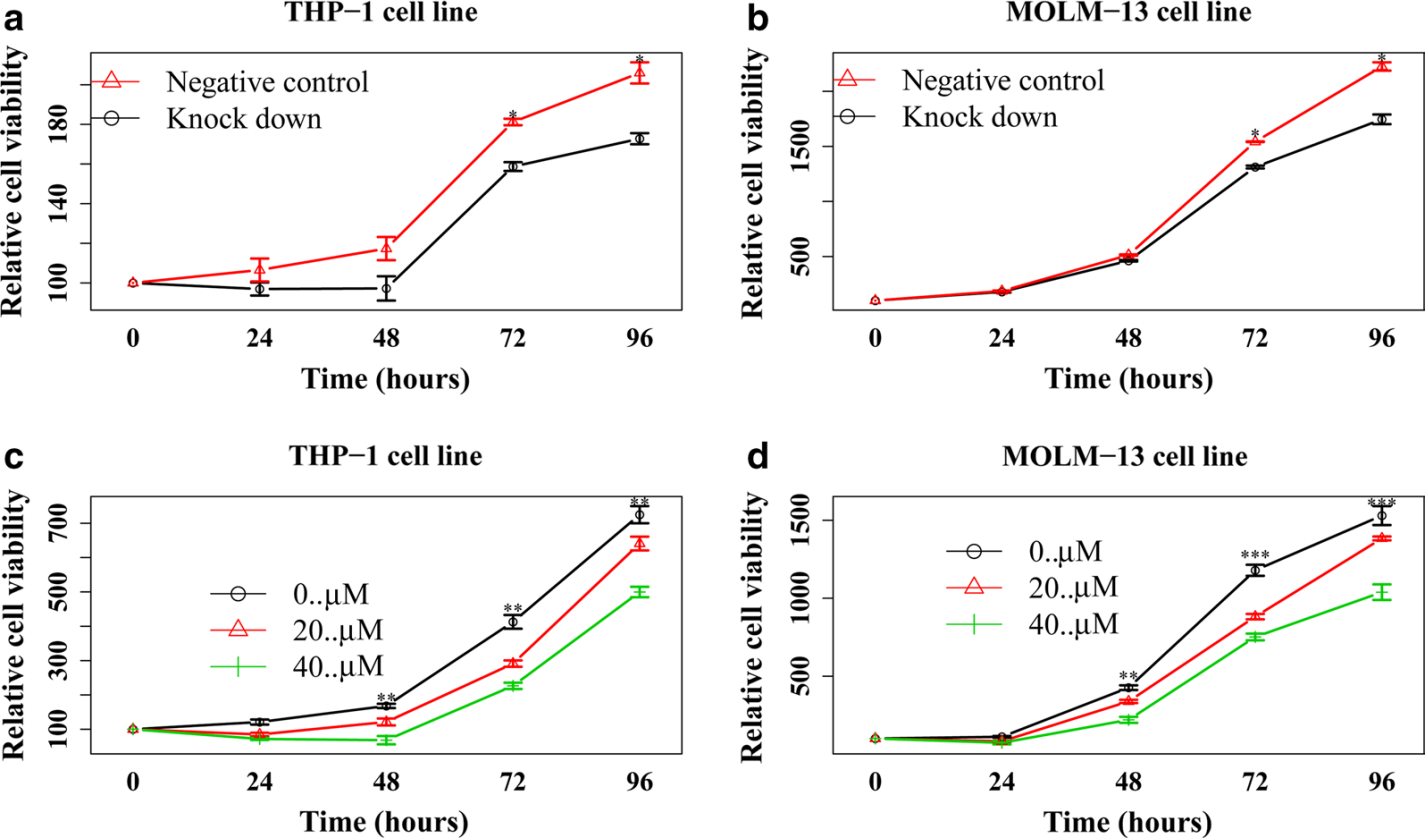
Knockdown of ACLY using the shRNA in THP-1 (**a**) and MOLM-13 (**b**) and the inhibitor SB-204990 in THP-1 (**c**) and MOLM-13 (**d**) cell lines

The implication of *ACLY* expression levels as an independent prognostic factor and its involvement in multiple pathways suggest that *ALCY* may be functionally important for maintaining the continuous proliferation of leukemia cells. To evaluate this possibility, firstly, we analyzed the expression levels of *ACLY* across hematopoietic cell types including acute myeloid leukemia (AML), chronic myeloid leukemia (CML), acute lymphoid leukemia (ALL) and myelodysplastic syndrome (MDS) in the BloodSpot web-based interface (http://www.bloodspot.eu, Additional file 1: Figure S4). As a result, we found that patients with t(11q23)/MLL abnormalities had the highest levels of *ACLY* expression among all kinds of hematopoietic cells (Additional file 1: Figure S4). In the subgroup analyses, *ACLY* expression was also higher in AML patients with t(11q23)/MLL abnormalities than in others such as healthy controls, AML patients with normal karyotype, t(8;21), t(15;17) and complex karyotypes, respectively. These results indicated that *ACLY* expression was predominant in AML blasts with t(11q23)/MLL abnormalities. However, the individual’s distribution of *ACLY* expression was very wide in patients with t(11q23)/MLL abnormalities, implying these patients obtained a diverse range of *ACLY* expressions. In order to test this result, secondly, we detected *ACLY* expression in a serial of leukemia cell lines with different genetic characteristics including Kasumi-1 with t(8;21), NB4 with t(15;17), MOLM-13 and THP-1 with t(11q23)/MLL, and other cell lines such as MV4-11, HL-60, OCI-AML3. As a result, THP-1 and MOLM-13 cell lines had the distinct expression levels of *ACLY*, although they had the same genetic background of t(11q23)/MLL abnormalities (Additional file 1: Figure S5). Of note, *ACLY* expression was the highest in THP-1 cell lines while the relatively low in MOLM13 cell lines. This expression pattern in proteins and mRNAs (Additional file 1: Figure S5) was in line with those exhibiting a diverse range of *ACLY* expression in patients with t(11q23)/MLL abnormalities. Of interest, there was a morsel of the inhibition effect of *ACLY* expression using *ACLY* inhibitor SB-204990 in KG-1 with TP53 mutation, while no difference was seen in IC50 values of the other cell lines (Additional file 1: Figure S5). At last, we measured the proliferation of MOLM-13 and THP-1 cells, using short hairpin RNA (shRNA) and small molecular inhibitors to silence *ACLY* expression. Knockdown expression of *ACLY* was confirmed by Western blot analysis after 48 h of shRNA treatment (Additional file 1: Figure S6). The knockdown of *ACLY* in the two leukemia cell lines resulted in growth arrest (Fig. 3a, b). In parallel, silencing *ACLY* expression with the inhibitor SB-204990 significantly reduced the proliferation of MOLM-13 and THP-1 leukemia cell lines (Fig. 3c, d). These results suggest that *ACLY* might have potential as a therapeutic target.

## Discussion

Metabolic reprogramming is generally regarded as a hallmark of cancer cells [20]. Since enhanced glycolysis was first reported in cancer cells by Otto Warburg, several studies have demonstrated that cancer cells also undergo aberrant TCA cycles and lipogenesis [21–26]. Recently, we also found that some fatty acids and TCA intermediates were associated with drug resistance [1, 3]. Importantly, enhanced glycolysis of glucose to lactate allows proliferating tumor cells to shunt various glycolytic intermediates into the fatty acid synthesis pathway [27]. Notably, *ACLY* as a lipogenesis enzyme links between TCA cycle and fatty acid synthesis pathway. Acetyl-CoA is the product of *ACLY,* serving as a vital building block for the endogenous biosynthesis of fatty acids, alongside other involvement in regulating nuclear and histone acetylation [9]. More importantly, it is now thought that *ACLY* expression may play an important role in both tumor metabolism and tumorigenesis [9, 28, 29]. Several studies have demonstrated that *ACLY* overexpression can serve as a prognostic factor in various solid tumors [10, 14, 28]. One possible reason is that the upregulated fatty acid synthesis caused by *ACLY* activity in tumor cells saturates membrane lipids, thereby leading to drug resistance and affecting therapy response [6].

In this study, we found low *ACLY* expression was associated with a high frequency of double allele *CEBPA* mutations and low levels of WBC (Table 1). It is well known that AML patients with the double allele *CEBPA* mutations are sensitive to conventional chemotherapy. These findings imply that *ACLY* expression might have prognostic significance for AML. Therefore, we further adopted a retrospective training-validation scheme to test the relationship between *ACLY* expression and OS in AML patients. In our training cohort, we found *ACLY* expressions were strongly associated with overall survival (Fig. 1). Because *ACLY* expression is also strongly associated with traditional clinical parameters with prognostic values, the correlation between *ACLY* expression and overall survival may be artificial. In order to exclude cytogenetic confounding variables, we further limited patients to the specific cytogenetic subgroups. Resultingly, we found *ACLY* expression was strongly associated with overall survival in AML, CI-AML and CN-AML patients, respectively (Fig. 1). This result supported the fact that the prognostic value of *ACLY* expressions may not be confounded by cytogenetic risk groups. Moreover, we repeated similar multivariate analyses, assuming other clinical parameters as confounding factors, such as age, WBC, cytogenetic risks and *FLT*3-ITD, *NPM1* and *CEBPA* gene mutations (Table 1). Even adjusting for these factors, low *ACLY* expression remained a significant prognostic predicting factor in the multivariate models (Table 2). Finally, the favorable survival of low *ACLY* expressing patients noted in our patient cohort was also validated in two independent cohorts of AML patients, following a similar methodology. Thus, we can confirm aberrant *ACLY* expression can serve as a predictor for survival in AML patients.

To further explore why low *ACLY* expression correlates with favorable survival in AML patients, we derived a gene expression signature using our three previously used datasets. Interestingly, downregulation of multiple pathways, alongside upregulation of 12 genes co-expressed in pediatric AML patients bearing inv(16) translocation, were seen in low *ACLY* expression patients (Additional file 1: Tables S4, S5). These results indicated that aberrant *ACLY* expression may play a critical role of the progression of AML. Therefore, we suppressed *ACLY* expression using shRNA and an inhibition. We found silencing *ACLY* expression does reduce the proliferation of leukemia blasts in vitro. This similar result was reported by Georgia Hatzivassiliou in tumor cells recently [30]. Thus, our results demonstrated that *ACLY* may promote leukemia growth with prognostic significance and can potentially be used as a novel drug target in the future.

## Conclusion

Building upon previously established work on using *ACLY* as a prognostic indicator in solid tumors, *ACLY* was found to have a similar significant prognostic use in AML patients in determining overall survival. Low *ACLY* expression levels corresponded to a better prognosis and overall survival, a prognostic indicator that remained even when accounting for potential confounding variables. *ACLY* further not only serves as a bridge between the TCA cycle and fatty acid synthesis, a critical step in the modified metabolic cycle of tumor cells, but also involves in multiple pathways. Disruption of *ACLY* either through silencing or knockdown showed growth arrest of AML cell lines, highlighting *ACLY*’s role as both important in the proliferation of AML and as a potential target for future drug therapies.

## Additional file

**Additional file 1.** Supplementary methods and data.
