# Supplementary material for: Low expression of ACLY associates with favorable prognosis in acute myeloid leukemia
Source: J Transl Med. 2019 May 10;17:149. doi: 10.1186/s12967-019-1884-5 (PMC6509777; doi:10.1186/s12967-019-1884-5)
Supplement: Supplementary file 1 — Additional file 1. Supplementary methods and data. [file 12967_2019_1884_MOESM1_ESM.docx]

**Low expression of *ACLY* associates with favorable prognosis in acute myeloid leukemia**

**Authors:**

Jinghan Wang^1,2,3^, Wenle Ye^2, 3^, Xiao Yan^3,4^, Qi Guo^5^, Qiuling Ma^6^, Fang Lin^1^, Jiansong Huang^2,3^, Jie Jin^1,2,3※^

**Affiliations:**

^1^Department of Hematology, The First Affiliated Hospital, Zhejiang University College of Medicine, Hangzhou, P.R. China.

^2^Institute of Hematology, Zhejiang University, Hangzhou, P.R. China.

^3^Key Laboratory of Hematologic Malignancies, Diagnosis and Treatment, Zhejiang, Hangzhou, PR China.

^4^Department of hematology, Qingdao Municipal hospital. Shandong, China.

^5^Department of Nephrology, The First Affiliated Hospital, Zhejiang University, Hangzhou, China

^6^Department of Hematology, the Second Affiliated Hospital of Henan University of Traditional Chinese Medicine. zhengzhou, China.

※Corresponding author: Jie Jin, Email: [jiej0503@zju.edu.cn](mailto:jiej0503@zju.edu.cn)

Tel: +86 571-87236898Fax: +86 571-87236702

**Patients and treatment protocols**

Clinical data were abstracted from medical records of AML patients in Zhejiang Institute of Hematology (ZIH), which is one of the hematologic centers in China. Between March 2010 and June 2017, 274 patients with detailed diagnoses and treatment information were enrolled in this study. WHO classification, conventional cytogenetic banding assay, and molecular analyses were performed as previously described in AML diagnosis^[^[^1^](#_ENREF_1)^]^. Cytogenetic risk groups of patients were classified as favorable, intermediate, and unfavorable risk according to the NCCN guideline^[^[^2^](#_ENREF_2)^]^. Favorable subgroups included t(8;21)/*AML1-ETO* and inv16/*CBFβ-MYH11*; adverse consisted of t(9;22), inv(3)/t(3;3), -5, -7, del(5q), del(7p), 11q23 and complex translocations; intermediate subtype contained cytogenetically normal and AML with other cytogenetic abnormalities. The protocols used for induction therapy in AML patients including HAA, homoharringtonine-based treatment (homoharringtonine 2 mg/m2 /day for 3 days, cytarabine 75 mg/m^2^ twice daily for 7 days, aclarubicin 12 mg/m^2^ daily for 7 days) regiment; DA, daunorubicin 45 mg/m^2^ daily for 3 days and cytarabine 100 mg/m^2^ daily for 7 days; IA, idarubicin 6-8 mg/m^2^ daily for 7 days and aclarubicin 20 mg/m^2^ daily for 5 days. In the consolidation therapy, younger patients were treated with a high-dose cytarabine-based chemotherapy^[^[^3^](#_ENREF_3)^]^. The chemotherapy consolidation for elderly patients was decided by the physicians in an individualized manner, as described previously^[^[^3^](#_ENREF_3)^]^. All of the subjects were well-informed about the study and provided written informed consent to participate in the study. The study was approved by the Institutional Review boards of the First Affiliated Hospital of Zhejiang University.

**Cytogenetic and Gene mutation analysis**

The BM samples of de novo AML patients were studied mostly by R-banding analysis. Chromosomal abnormalities were described according to the International System for Human Cytogenetic Nomenclature^[^[^4^](#_ENREF_4)^]^. DNA and RNA samples of AML patients were obtained from mononuclear cells isolated by Ficoll gradient centrifugation from bone marrow samples at primary diagnosis. Gene mutations of *NPM1*, *FLT3*ITD and *CEBPA* were analyzed by whole-gene sequencing as previously described^[^[^5^](#_ENREF_5)^]^. RNA samples were used to determine *PMLRARA, AML1ETO*, and *CBFβMYH11* fusion genes by reverse transcription polymerase chain reaction (RT-PCR). All PCR products were directly sequenced with both forward and reverse primers to ensure quality. All sequence data were read using Chromas version 2.22 software.

**Cell cultures**

The AML cell line THP-1, Kasumi-1, NB4 and HL-60 were purchased from the Shanghai Cell Culture Institute (Shanghai, China)，MV4-11, MOLM-13, OCI-AML3 cell line were gifts from Professor Ravi Bhatia (City of Hope National Medical Center, Duarte, CA). The THP-1, Kasumi-1, NB4 and HL-60 were cultured in RPMI-1640 (Gibco, Billings, MT, USA) supplemented with 10% fetal bovine serum (FBS, Gibco Billings, MT, USA), MV4-11 and MOLM-13 were cultured in IMDM medium (Gibco, Billings, MT, USA) supplemented with 10% fetal bovine serum (Gibco). All cell lines and primary cells were maintained at 37°C with 5% CO_2_.

Human ACLY shRNA lentivirus plasmid was obtained from Genecopoeia (USA). The targeting sequences of each shRNA are shown in Table S2. Lentiviral production in 293T cells was done using standard protocols. Transfection mix containing viral variant, pMD2G, and psPAX vectors were combined with TE buffer, H_2_O, CaCl_2_, and HeBS, incubated for 5 min and added to subconfluent 293T cells in 15 cm plates. Subsequently, the cells were incubated at 37°C with 5% CO2 for 6h. Media was replaced and fresh media was added to the cells. Again, the 293T cells were incubatedat 37°C with 5% CO2 for 72h. Media was then collected, filtered using a 0.45 mM filter and frozen. Viral supernatant combined with 4 mg/ml of puromycin with serum free media was added to THP-1 and MV4-11 cells with Proper density. Media was changed after 16 hand cells were analyzed 72 h after knockdown experiment.

**Cell Viability Assay**

ColorimetricCellTiter 96 AQueous One Solution Cell Proliferation Assay (MTS assay, Promega, Madison, WI, USA) was used to measure cell proliferation. AML cells were seeded in 96-well culture plates at proper density (5×10^4^ viable cells). AML cells which transfected by sh-ACLY and sh-NC Lentivirus plasmidwere seeded into 96-well culture plates at 1×10^4^cells/ml for 0, 1, 2, 3, 4days, respectively. Cells were treated with ACLY inhibitor SB-204990 (Bioscience UK) of different concentrations, respectively, for 24h, 48h, 72h and 96h; at the same time, equal volume of DMSO was added to cells as negative control. MTS assay was used to determine the cytotoxicity. The absorbance at 490nm was measured for each well. Cell-line experiments were triplicated.

**Western blot analyses**

Cells were washed twice with 1xPBS and lysed in 1x radioimmunoprecipitation assay (RIPA) buffer. The protein concentration of the samples was determined by a bicinchoninic acid (BCA) Protein Assay kit (Pierce, Rockford, IL, USA). Protein lysates were separated on SDS–PAGE and transferred onto PVDF membranes. Membranes were blocked for 1 h at room temperature with 5% skimmed milk in TBS-T (10 mMTris–HCl pH 8.0, 150 mMNaCl, 0.1% Tween-20) and incubated with primary antibodies at 1:1000 dilution overnight at 4 ℃. Membranes were then washed three times with TBS-T buffer, incubated with HRP-conjugated secondary antibody at 1:5000 dilution (KPL, Baltimore, MD, USA) for 1 h at room temperature, and washed three times again with TBS-T buffer. The results were analyzed using an ECL kit (Amersham, Little Chalfont, UK) and imagine lab software.

Reference

1. Wang JH, Chen WL, Li JM, Wu SF, Chen TL, Zhu YM, Zhang WN, Li Y, Qiu YP, Zhao AH, Mi JQ, Jin J, Wang YG, Ma QL, Huang H, Wu DP, Wang QR, Yan XJ, Yan JS, Li JY, Wang S, Huang XJ, Wang BS, Jia W, Shen Y, Chen Z, Chen SJ, Prognostic significance of 2-hydroxyglutarate levels in acute myeloid leukemia in China, Proc Natl Acad Sci U S A, 2013, 110(42): 17017-22

2. O'Donnell MR, Abboud CN, Altman J, Appelbaum FR, Arber DA, Attar E, Borate U, Coutre SE, Damon LE, Goorha S, Lancet J, Maness LJ, Marcucci G, Millenson MM, Moore JO, Ravandi F, Shami PJ, Smith BD, Stone RM, Strickland SA, Tallman MS, Wang ES, Naganuma M, Gregory KM, Acute myeloid leukemia, J Natl Compr Canc Netw, 2012, 10(8): 984-1021

3. Ma QL, Wang JH, Wang YG, Hu C, Mu QT, Yu MX, Wang L, Wang DM, Yang M, Yin XF, Chen FF, Lu SS, Chen J, Zhu ZJ, Chen SJ, Jin J, High IDH1 expression is associated with a poor prognosis in cytogenetically normal acute myeloid leukemia, Int J Cancer, 2014:

4. Brothman AR, Persons DL, Shaffer LG, Nomenclature evolution: Changes in the ISCN from the 2005 to the 2009 edition, Cytogenet Genome Res, 2009, 127(1): 1-4

5. Chen WL, Wang JH, Zhao AH, Xu X, Wang YH, Chen TL, Li JM, Mi JQ, Zhu YM, Liu YF, Wang YY, Jin J, Huang H, Wu DP, Li Y, Yan XJ, Yan JS, Li JY, Wang S, Huang XJ, Wang BS, Chen Z, Chen SJ, Jia W, A distinct glucose metabolism signature of acute myeloid leukemia with prognostic value, Blood, 2014, 124(10): 1645-54

Table S1. Primer and sequence of mRNA qRT-PCR

| primer | Forward | Reverse |
| --- | --- | --- |
| *ABL1* | TGGAGATAACACTCTAAGCATAACTAAAGGT | CCATTTTTGGTTTGGGCTTCACACCATT |
| *ACLY* | ATCGGTTCAAGTATGCTCGGG | GACCAAGTTTTCCACGACGTT |

Table S2. shRNA Target Sequences

| **Clone Name** | **Symbol** | **Length** | **Target Sequence** |
| --- | --- | --- | --- |
| CSHCTR001-1-LVRU6GP(OSNEG20) | NC | 19 | gcttcgcgccgtagtctta |
| HSH011821-1-LVRU6GP(OS210433) | ACLY | 19 | gaatcggttcaagtatgct |

Table S3. Characteristics of AML patients by high and low ACLY expression from the TCGA cohort

| Variables | Low expression | High expression | P value |
| --- | --- | --- | --- |
| Number | 49 | 148 |  |
| Age, median(range),years | 57.00 [38.00, 65.00] | 57.00 [45.00, 67.00] | 0.315 |
| Male, n(%) | 32 (65.3) | 74 (50.0) | 0.07 |
| WBC, median(range),×10^9/L^1^ | 5.50 [2.10, 32.40] | 21.05 [5.33, 60.32] | <0.001 |
| Percent BM blast, median(range),%^4^ | 71.00 [56.00, 90.00] | 73.00 [53.00, 85.00] | 0.297 |
| FAB classification, n(%)^5^ |  |  | 1.000 |
| M0 | 6 (12.2) | 11 ( 7.4) |  |
| M1 | 13 (26.5) | 33 (22.3) |  |
| M2 | 13 (26.5) | 31 (20.9) |  |
| M3 | 12 (24.5) | 7 ( 4.7) |  |
| M4 | 2 ( 4.1) | 39 (26.4) |  |
| M5 | 1 ( 2.0) | 21 (14.2) |  |
| M6 | 1 ( 2.0) | 2 ( 1.4) |  |
| M7 | 1 ( 2.0) | 2 ( 1.4) |  |
| Unclassified | 0 ( 0.0) | 2 ( 1.4) |  |
| Karyotype risk, n(%) |  |  | 0.118 |
| Favorable | 12 (24.5) | 24 (16.2) |  |
| Intermediate | 23 (46.9) | 91 (61.5) |  |
| Unfavorable | 0 ( 0.0) | 5 ( 3.4) |  |
| N.D. | 14 (28.6) | 28 (18.9) |  |
| Genes mutations, n(%) |  |  |  |
| *FLT3*-ITD | 7 (14.3) | 48 (32.4) | 0.016 |
| *NPM1* | 5 (10.2) | 49 (33.1) | 0.002 |
| *CEBPA^DM6^* | 2 ( 4.1) | 3 ( 2.0) | 0.389 |
| *DNMT3A* | 6 (12.2) | 44 (29.7) | 0.014 |
| *IDH1* | 5 (10.2) | 14 ( 9.5) | 1 |
| *IDH2* | 7 (14.3) | 12 ( 8.1) | 0.262 |
| BMT | 25 (51.0) 62 (41.9) 0.320 | | |

Abbreviations:^1^WBC,white blood cell; ^2^HB, hemoglobin; ^3^PLT, platelet counts; ^4^BM, bone marrow; ^5^FAB, French–American–British classification systems;^6^DM: Double-allele. BMT, bone marrow transplantation.

Table S4. Univariate and multivariate analysis of AML patients from TCGA dataset for overall survival

| Variables | Univariate analysis | | Multivariate analysis | |  |
| --- | --- | --- | --- | --- | --- |
|  | P value | HR (95%CI) | P value | HR (95%CI) |  |
| ACLY (High vs. Low) | 0.014 | 1.684(1.11,2.553) | 0.032 | 1.625(1.044,2.531) | |
| Age | <0.001 | 1.04(1.027,1.054) | 0.001 | 1.026(1.011,1.042) | |
| WBC | 0.122 | 1.003(0.999,1.006) | 0.612 | 1.001(0.997,1.005) | |
| Cytogenetic risks |  |  |  |  |  |
| Intermediate vs Favorable | <0.001 | 3.202(1.742,5.883) | <0.001 | 3.536(1.755,7.125) | |
| Poor vs Favorable | <0.001 | 4.561(2.346,8.865) | <0.001 | 7.743(3.655,16.406) | |
| Not detemine vs Favorable | 0.001 | 6.851(2.186,21.468) | <0.001 | 9.096(2.8,29.553) | |
| Gene mutations (mutation vs. wild-type) | |  |  |  |  |
| *FLT3*-ITD | 0.146 | 1.325(0.907,1.937) | 0.035 | 1.646(1.035,2.618) | |
| *NPM1* | 0.303 | 1.219(0.836,1.779) | 0.243 | 0.74(0.446,1.227) | |
| *CEBPA^DM2^* | 0.436 | 0.634(0.202,1.995) | 0.441 | 1.639(0.467,5.755) | |
| *DNMT3A* | 0.013 | 1.616(1.106,2.361) | 0.519 | 1.148(0.755,1.746) | |
| BMT | 0.001 | 0.542(0.382,0.771) | <0.001 | 0.421(0.272,0.65) | |

Abbreviations: WBC,white blood cell; DM: Double-allele. BMT, bone marrow transplantation.

Table S5. Genes expression associated with low ACLY expression by metaanalyis.

| Genes | Low group | meta.FDR |
| --- | --- | --- |
| GNS | Down | 0.039509 |
| KCTD12 | Down | 0.000793 |
| DR1 | Down | 0.021294 |
| TM9SF2 | Down | 0.000518 |
| ARPC1B | Down | 0.002474 |
| VCAN | Down | 0.028897 |
| NDFIP1 | Down | 0.005836 |
| NPTN | Down | 0.01784 |
| PROSC | Down | 0.009416 |
| CTSZ | Down | 0.004185 |
| CPNE3 | Down | 0.005789 |
| PGAM1 | Down | 0.033474 |
| CREBL2 | Down | 6.25E-05 |
| WSB2 | Down | 0.005836 |
| ATP6V1E1 | Down | 0.000518 |
| HEXB | Down | 0.010718 |
| SORT1 | Down | 0.036561 |
| FAM120A | Down | 0.000926 |
| NMT1 | Down | 0.000518 |
| RAB31 | Down | 0.007103 |
| FBXL5 | Down | 0.012336 |
| ATP6V1A | Down | 5.44E-18 |
| CX3CR1 | Down | 0.000753 |
| SLC25A24 | Down | 0.001242 |
| MEGF9 | Down | 0.005789 |
| DYNC1I2 | Down | 0.000544 |
| PPT1 | Down | 0.027522 |
| GPD1L | Down | 0.009326 |
| VNN1 | Down | 0.019005 |
| SARS | Down | 0.008673 |
| SCP2 | Down | 0.048284 |
| PRKAR1A | Down | 0.001684 |
| FGL2 | Down | 0.025479 |
| ARPC5 | Down | 0.000892 |
| GHITM | Down | 0.007558 |
| HTATIP2 | Down | 0.000667 |
| CLTC | Down | 0.000793 |
| CBX1 | Down | 0.00222 |
| SIDT2 | Down | 0.032098 |
| EIF4E2 | Down | 0.018893 |
| ARL6IP5 | Down | 0.002679 |
| METTL9 | Down | 0.000107 |
| ACTR2 | Down | 0.002649 |
| ORMDL2 | Down | 0.006851 |
| GNPDA1 | Down | 0.000107 |
| PECAM1 | Down | 0.000667 |
| PDCD6IP | Down | 0.03364 |
| PRDX3 | Down | 0.000929 |
| OSBPL11 | Down | 0.004036 |
| GRB2 | Down | 0.021024 |
| ITGAM | Down | 0.048967 |
| ATP6V0E1 | Down | 0.000932 |
| MAPK1 | Down | 0.006564 |
| IARS2 | Down | 0.003254 |
| ZMPSTE24 | Down | 5.44E-18 |
| ACTR3 | Down | 5.44E-18 |
| CD1D | Down | 0.024286 |
| OPN3 | Down | 0.00074 |
| CD180 | Down | 0.021294 |
| CD93 | Down | 0.02477 |
| TOR1AIP1 | Down | 0.02049 |
| NADK | Down | 0.033474 |
| SEC23B | Down | 0.002214 |
| FH | Down | 5.44E-18 |
| COQ2 | Down | 5.44E-18 |
| CD36 | Down | 0.018556 |
| TMEM33 | Down | 0.007491 |
| SDHB | Down | 0.000219 |
| CPPED1 | Down | 0.004073 |
| G6PD | Down | 0.032027 |
| CCR2 | Down | 0.004278 |
| AIDA | Down | 6.25E-05 |
| ME2 | Down | 0.000105 |
| SKAP2 | Down | 0.048585 |
| FUCA1 | Down | 0.016347 |
| YWHAB | Down | 0.000954 |
| BCL2L13 | Down | 0.007016 |
| ARCN1 | Down | 0.000793 |
| C1GALT1C1 | Down | 0.000753 |
| FAR2 | Down | 0.039509 |
| PAK1 | Down | 0.01615 |
| RSU1 | Down | 0.000518 |
| CCDC53 | Down | 0.000954 |
| M6PR | Down | 0.002437 |
| PSMD7 | Down | 0.012222 |
| ITFG1 | Down | 0.026597 |
| MCOLN1 | Down | 0.007759 |
| ENOX2 | Down | 0.000793 |
| NSF | Down | 6.25E-05 |
| CALML4 | Down | 0.005629 |
| APAF1 | Down | 0.011304 |
| NDUFB3 | Down | 0.026486 |
| TCEB2 | Down | 0.025805 |
| CYB5R1 | Down | 0.032355 |
| OSTM1 | Down | 0.000667 |
| PSMA7 | Down | 0.047565 |
| NRGN | Down | 0.006641 |
| NBR1 | Down | 5.44E-18 |
| HK1 | Down | 0.003661 |
| GLRX | Down | 0.019617 |
| ST8SIA4 | Down | 0.000386 |
| PDHB | Down | 0.000381 |
| MCTS1 | Down | 0.006224 |
| PSMB3 | Down | 0.008015 |
| GSN | Down | 0.047708 |
| CPT2 | Down | 0.000275 |
| MLX | Down | 0.000237 |
| GCA | Down | 0.040659 |
| SNX3 | Down | 0.022319 |
| CIDEB | Down | 0.002429 |
| MYL12B | Down | 0.004659 |
| SDHD | Down | 0.000105 |
| PPA2 | Down | 0.002994 |
| FAM65B | Down | 0.005196 |
| ENTPD1 | Down | 0.024733 |
| PCTP | Down | 0.000667 |
| UCP2 | Down | 0.031778 |
| SNX6 | Down | 0.000107 |
| NDUFB5 | Down | 0.002862 |
| PSMB2 | Down | 0.00108 |
| PIGK | Down | 0.048967 |
| CCT5 | Down | 0.003661 |
| NDUFS2 | Down | 0.001684 |
| OAZ1 | Down | 0.044028 |
| MSL1 | Down | 5.44E-18 |
| PTGES3 | Down | 0.033474 |
| EIF2S2 | Down | 0.046124 |
| NDUFS7 | Down | 0.001392 |
| ATP5E | Down | 0.003308 |
| ACLY | Down | 5.44E-18 |
| RAB7A | Down | 0.015824 |
| ZBTB38 | Down | 0.003708 |
| IDH1 | Down | 0.001286 |
| ACTR10 | Down | 0.014043 |
| ATP5F1 | Down | 0.000753 |
| ADAP2 | Down | 0.026363 |
| SRI | Down | 0.037427 |
| ERLIN2 | Down | 0.002088 |
| EPB41L2 | Down | 0.000645 |
| TBC1D22A | Down | 0.033474 |
| SLC35A1 | Down | 5.44E-18 |
| TMED10 | Down | 0.001291 |
| PSMD9 | Down | 0.01171 |
| TALDO1 | Down | 0.018711 |
| ALDH3A2 | Down | 0.036346 |
| PSMA5 | Down | 0.000237 |
| MRPS28 | Down | 0.004448 |
| HDAC9 | Down | 0.001209 |
| TUBA4A | Down | 0.017795 |
| VTI1B | Down | 0.045824 |
| PCYOX1L | Down | 0.008217 |
| NAGK | Down | 0.003114 |
| VPS35 | Down | 0.002352 |
| TBC1D1 | Down | 0.006256 |
| NDUFA6 | Down | 0.007103 |
| CD300A | Down | 0.02212 |
| AKAP7 | Down | 2.00E-04 |
| SURF1 | Down | 0.024773 |
| BAG4 | Down | 0.016503 |
| AGPAT3 | Down | 0.0222 |
| PPCS | Down | 0.000793 |
| GABARAPL2 | Down | 0.005627 |
| IDH3A | Down | 0.000929 |
| DTX4 | Down | 0.007103 |
| TINF2 | Down | 0.035068 |
| CRK | Down | 0.007103 |
| RER1 | Down | 0.028945 |
| PSMD8 | Down | 0.032825 |
| TRAPPC3 | Down | 0.010718 |
| VCP | Down | 0.044165 |
| NECAP2 | Down | 0.000458 |
| BLVRA | Down | 0.013033 |
| SLC35A5 | Down | 0.00088 |
| PAK2 | Down | 0.015185 |
| METTL7A | Down | 0.000222 |
| VPS26A | Down | 0.024281 |
| FIBP | Down | 0.045242 |
| TMEM9B | Down | 0.039485 |
| DNAJC13 | Down | 0.00553 |
| MEF2C | Down | 0.000518 |
| CISD1 | Down | 0.021824 |
| HIGD1A | Down | 0.000458 |
| ARF6 | Down | 0.044364 |
| WDR7 | Down | 5.44E-18 |
| TBL1X | Down | 0.032077 |
| IMPA2 | Down | 0.040578 |
| TRIP4 | Down | 0.00122 |
| PCMT1 | Down | 5.44E-18 |
| PGLS | Down | 0.033474 |
| HMGN4 | Down | 0.034086 |
| BECN1 | Down | 0.00471 |
| EIF2S1 | Down | 0.001242 |
| BTK | Down | 0.003885 |
| TDRD7 | Down | 0.000386 |
| COPB2 | Down | 0.015992 |
| SRD5A1 | Down | 0.004957 |
| TPST2 | Down | 0.005836 |
| UBE2A | Down | 0.008091 |
| BIN2 | Down | 0.032544 |
| TRAFD1 | Down | 0.049271 |
| SEC23IP | Down | 0.003308 |
| PARK7 | Down | 0.006279 |
| ANKRD40 | Down | 0.000714 |
| TMX1 | Down | 0.002701 |
| EIF3K | Down | 0.03609 |
| PPBP | Down | 0.000518 |
| CRLF3 | Down | 0.024189 |
| PSMD14 | Down | 0.027851 |
| QDPR | Down | 0.010493 |
| PSME1 | Down | 0.025344 |
| ELP4 | Down | 0.004689 |
| MRPL34 | Down | 0.011638 |
| CYBRD1 | Down | 0.009616 |
| MRPS14 | Down | 0.036561 |
| PSMD1 | Down | 0.004077 |
| SNRPG | Down | 0.004659 |
| ASAP1 | Down | 0.010518 |
| PSMA2 | Down | 0.038699 |
| COPS6 | Down | 0.000667 |
| TEX2 | Down | 0.00122 |
| FKBP3 | Down | 0.0038 |
| ALDH9A1 | Down | 0.012967 |
| GLRX2 | Down | 0.005836 |
| STARD7 | Down | 0.000105 |
| IRF2 | Down | 6.25E-05 |
| CDC123 | Down | 0.03609 |
| ARHGAP19 | Down | 0.000843 |
| SMAD2 | Down | 0.013849 |
| PPP2R5C | Down | 0.004031 |
| PPP1CC | Down | 0.000954 |
| UQCR11 | Down | 0.005836 |
| C11ORF24 | Down | 0.041293 |
| BLNK | Down | 0.045238 |
| MDH1 | Down | 0.000932 |
| DCTN2 | Down | 0.003973 |
| ATP5C1 | Down | 0.014234 |
| NDUFS1 | Down | 0.018711 |
| JTB | Down | 0.024871 |
| MSL3 | Down | 0.00924 |
| PANK2 | Down | 0.000107 |
| PPID | Down | 0.003012 |
| NMI | Down | 0.048046 |
| SNX17 | Down | 0.031627 |
| CACYBP | Down | 0.010826 |
| LSM6 | Down | 0.002641 |
| GPN3 | Down | 0.000107 |
| EGLN1 | Down | 0.027163 |
| TFDP1 | Down | 0.0198 |
| IFIT5 | Down | 0.000954 |
| MPPE1 | Down | 0.046802 |
| RPE | Down | 0.001276 |
| UNC50 | Down | 0.000954 |
| SPCS3 | Down | 0.02001 |
| MICAL2 | Down | 0.009377 |
| COX5A | Down | 0.02477 |
| NDUFAF1 | Down | 0.022482 |
| SNAPC5 | Down | 0.000222 |
| TMCO6 | Down | 0.008732 |
| MRPL15 | Down | 0.038636 |
| GLT8D1 | Down | 0.001297 |
| TMEM184C | Down | 0.022876 |
| FDFT1 | Down | 0.001286 |
| TMCO1 | Down | 0.001455 |
| ZNF318 | Down | 0.000458 |
| ACAT1 | Down | 0.000458 |
| CANT1 | Down | 0.001124 |
| BRCC3 | Down | 0.00553 |
| RGS10 | Down | 0.00677 |
| NDUFB1 | Down | 0.007103 |
| SERPINB9 | Down | 0.032125 |
| POLR2G | Down | 0.009936 |
| PSMB4 | Down | 0.005836 |
| TSNAX | Down | 0.000667 |
| AHSA1 | Down | 0.017283 |
| DERA | Down | 0.039509 |
| COX7A2 | Down | 0.007417 |
| CDIPT | Down | 0.000753 |
| DHRS7B | Down | 0.000753 |
| ATF1 | Down | 0.035795 |
| SNRPD3 | Down | 0.048284 |
| STX7 | Down | 0.000807 |
| ARFIP1 | Down | 0.002869 |
| ASB13 | Down | 0.000915 |
| ATRN | Down | 0.000929 |
| PSMB6 | Down | 0.036396 |
| SUCLG1 | Down | 0.000932 |
| UBE3C | Down | 0.000932 |
| TRAF3IP3 | Down | 0.024546 |
| KPNA1 | Down | 0.025212 |
| CHCHD3 | Down | 0.018194 |
| ANP32E | Down | 0.00237 |
| CKLF | Down | 0.026486 |
| ENOPH1 | Down | 0.005836 |
| RB1 | Down | 0.001248 |
| SMC1A | Down | 0.005099 |
| NDUFS6 | Down | 0.024608 |
| HEATR3 | Down | 0.001276 |
| SUCLA2 | Down | 0.006071 |
| IL17RA | Down | 0.006224 |
| C5 | Down | 0.021817 |
| PSMG2 | Down | 0.020697 |
| CLCN3 | Down | 0.001684 |
| FGD2 | Down | 0.046699 |
| GOLGA5 | Down | 0.019642 |
| TDP2 | Down | 0.002196 |
| VRK2 | Down | 0.004031 |
| CNDP2 | Down | 0.00243 |
| AIMP2 | Down | 0.002679 |
| POLD3 | Down | 0.002728 |
| VPS41 | Down | 0.002925 |
| AURKAIP1 | Down | 0.009154 |
| STAM2 | Down | 0.020108 |
| NDUFA2 | Down | 0.018778 |
| COPZ1 | Down | 0.02212 |
| TRADD | Down | 0.003661 |
| NUP37 | Down | 0.003661 |
| LMO2 | Down | 0.003726 |
| EIF2B1 | Down | 0.02473 |
| PSMF1 | Down | 0.004026 |
| SETD3 | Down | 0.018711 |
| IFIH1 | Down | 0.046729 |
| FYB | Down | 0.033474 |
| RHOT1 | Down | 0.004991 |
| PNPO | Down | 0.004991 |
| PF4 | Down | 0.007912 |
| LACTB2 | Down | 0.005265 |
| SP1 | Down | 0.01287 |
| FPGT | Down | 0.00553 |
| APOL3 | Down | 0.015824 |
| SIPA1 | Down | 0.04078 |
| PSRC1 | Down | 0.005789 |
| MGAT2 | Down | 0.017715 |
| DAP3 | Down | 0.00605 |
| GLOD4 | Down | 0.042555 |
| GTF2H5 | Down | 0.03609 |
| PLCL2 | Down | 0.008058 |
| CEP55 | Down | 0.008091 |
| SNAP23 | Down | 0.039288 |
| CCT3 | Down | 0.020108 |
| MRPL18 | Down | 0.008639 |
| COMMD10 | Down | 0.008732 |
| PSMA4 | Down | 0.019698 |
| CYB5B | Down | 0.044655 |
| IMMT | Down | 0.033878 |
| EDEM3 | Down | 0.021294 |
| CYB5R4 | Down | 0.025036 |
| AKR7A2 | Down | 0.010718 |
| NAIP | Down | 0.037427 |
| ZNF143 | Down | 0.011415 |
| ETFA | Down | 0.011476 |
| KLHL12 | Down | 0.012967 |
| PIK3CD | Down | 0.014969 |
| NARS | Down | 0.013171 |
| PTGS1 | Down | 0.013544 |
| G3BP1 | Down | 0.014286 |
| PAPOLA | Down | 0.03294 |
| SLC25A40 | Down | 0.02212 |
| NDUFB6 | Down | 0.049497 |
| LSM5 | Down | 0.018995 |
| SDHA | Down | 0.019359 |
| C5ORF22 | Down | 0.019642 |
| EEA1 | Down | 0.019658 |
| GIMAP6 | Down | 0.032969 |
| STAMBP | Down | 0.022876 |
| ATP6V1D | Down | 0.039509 |
| GPN2 | Down | 0.02254 |
| HDDC2 | Down | 0.023016 |
| H2AFZ | Down | 0.024473 |
| ADCY7 | Down | 0.032825 |
| SMAP1 | Down | 0.024608 |
| IDE | Down | 0.027377 |
| TXNL4A | Down | 0.027789 |
| DDX23 | Down | 0.032544 |
| SNF8 | Down | 0.032777 |
| C17ORF62 | Down | 0.033474 |
| CHMP5 | Down | 0.034536 |
| WIPF1 | Down | 0.035811 |
| MKKS | Down | 0.035962 |
| TMPO | Down | 0.036223 |
| ECHDC1 | Down | 0.036396 |
| RMI1 | Down | 0.036488 |
| NDUFAB1 | Down | 0.036561 |
| ADCK2 | Down | 0.038461 |
| HPRT1 | Down | 0.040308 |
| RCAN1 | Down | 0.046542 |
| SLC25A5 | Down | 0.047573 |
| PPM1G | Down | 0.047661 |
| TST | Down | 0.04918 |
| MMD | Down | 0.049275 |
| SLC4A1AP | Down | 0.049285 |
| PDE4D | Up | 0.037531 |
| STMN3 | Up | 0.032098 |
| CD99 | Up | 0.028945 |
| VPS13D | Up | 0.03294 |
| TCEAL4 | Up | 0.047593 |
| ADARB1 | Up | 0.005394 |
| CSNK1E | Up | 0.004318 |
| ELL | Up | 0.011363 |
| ZBTB1 | Up | 0.042391 |
| SPRY2 | Up | 0.026044 |
| DNAH3 | Up | 0.013279 |
| SENP2 | Up | 0.003012 |
| RPL36A | Up | 0.01093 |
| CD81 | Up | 0.048385 |
| RPGR | Up | 0.040659 |
| GPR153 | Up | 0.041303 |
| CDK16 | Up | 0.004396 |
| TGIF1 | Up | 0.020467 |
| MTMR9 | Up | 0.038701 |
| ZNF395 | Up | 0.000275 |
| ABCA1 | Up | 0.020667 |
| DUSP10 | Up | 0.039686 |
| DENND3 | Up | 0.003185 |
| PTPRO | Up | 0.026165 |
| SAMD4B | Up | 0.000633 |
| PLEKHA5 | Up | 0.024751 |
| AMPD2 | Up | 0.000645 |
| CLCN6 | Up | 0.040174 |
| INO80D | Up | 0.015762 |
| EIF2AK3 | Up | 0.014234 |
| TSPAN32 | Up | 0.020108 |
| BMP1 | Up | 0.031444 |
| DCTN6 | Up | 0.00145 |
| SEMA3F | Up | 0.018631 |
| TPM4 | Up | 0.00074 |
| RECK | Up | 0.006462 |
| ANKRD11 | Up | 0.005789 |
| BAZ2A | Up | 0.038461 |
| ARID5A | Up | 0.000603 |
| MARCKSL1 | Up | 0.013988 |
| ZSCAN18 | Up | 0.033474 |
| JAK3 | Up | 0.025212 |
| GNA15 | Up | 0.024345 |
| ME3 | Up | 0.024189 |
| EBLN2 | Up | 0.004214 |
| CTDP1 | Up | 0.017495 |
| TRAF5 | Up | 0.009645 |
| HMBOX1 | Up | 0.005836 |
| SFSWAP | Up | 0.005879 |
| CYP1A2 | Up | 0.018573 |
| CDK9 | Up | 0.00074 |
| CXORF40A | Up | 0.000929 |
| DNM1 | Up | 0.003973 |
| WDR74 | Up | 0.011286 |
| ZNF839 | Up | 0.007912 |
| HYMAI | Up | 0.023022 |
| ADA | Up | 0.013171 |
| ZNF254 | Up | 0.017667 |
| GNL1 | Up | 0.009403 |
| UBR5 | Up | 0.002994 |
| ZNF331 | Up | 0.010826 |
| CXORF40B | Up | 0.001286 |
| PHTF1 | Up | 0.002315 |
| RHOH | Up | 0.021145 |
| AGRN | Up | 0.007504 |
| IFT122 | Up | 0.005703 |
| RBBP6 | Up | 0.000844 |
| ATXN2L | Up | 0.00061 |
| AUTS2 | Up | 0.009936 |
| BRD1 | Up | 0.013171 |
| FLJ10038 | Up | 0.00431 |
| LOC647070 | Up | 0.010111 |
| KDM3A | Up | 0.027789 |
| HMGXB3 | Up | 0.00231 |
| ARHGEF7 | Up | 0.046699 |
| POFUT2 | Up | 0.000222 |
| ZNF337 | Up | 0.010799 |
| FBRS | Up | 0.032825 |
| ATF4 | Up | 0.000107 |
| CHD4 | Up | 0.004026 |
| WDR48 | Up | 0.004923 |
| ZBTB5 | Up | 0.015246 |
| ANKLE2 | Up | 0.012807 |
| ZNF432 | Up | 0.003271 |
| NOP2 | Up | 0.023452 |
| PARP6 | Up | 0.043551 |
| ZBTB17 | Up | 0.006157 |
| LRRC37A3 | Up | 0.046802 |
| IP6K2 | Up | 5.44E-18 |
| TAF9B | Up | 0.032138 |
| IL11RA | Up | 0.000518 |
| ANKRD10 | Up | 0.002437 |
| ALMS1 | Up | 0.006375 |
| SOX12 | Up | 0.029537 |
| EPM2AIP1 | Up | 0.026143 |
| ZMYM5 | Up | 0.007103 |
| JARID2 | Up | 0.002214 |
| LRP5L | Up | 0.000518 |
| RNMT | Up | 0.039509 |
| NSUN6 | Up | 0.001202 |
| RRN3P1 | Up | 0.032544 |
| TTF1 | Up | 0.036396 |
| REV3L | Up | 0.000793 |
| BRD2 | Up | 0.000107 |
| POLR1C | Up | 0.023058 |
| POU2F1 | Up | 0.026586 |
| HIC2 | Up | 0.014109 |
| KLHL24 | Up | 0.024275 |
| ZNF292 | Up | 0.038076 |
| NKTR | Up | 0.038073 |
| ABL1 | Up | 0.003841 |

Down and Up represent expression values of genes are respectively low and high in low ALCY group.

Table S6. Aberrant pathways involving in low ACLY in AML patients

| Name | p-value |
| --- | --- |
| Oxidative phosphorylation | 1.33E-14 |
| Proteasome | 1.76E-12 |
| Parkinson's disease | 4.33E-12 |
| Huntington's disease | 8.68E-12 |
| Alzheimer's disease | 2.79E-11 |
| Citrate cycle (TCA cycle) | 5.75E-11 |
| Carbon metabolism | 2.94E-10 |
| Metabolic pathways | 3.26E-10 |
| Non-alcoholic fatty liver disease (NAFLD) | 5.92E-10 |
| Citrate cycle (TCA cycle, Krebs cycle) | 1.06E-09 |
| Citrate cycle, second carbon oxidation, 2-oxoglutarate => oxaloacetate | 1.47E-08 |
| Endocytosis | 6.23E-06 |
| Pyruvate metabolism | 3.88E-05 |
| Pentose phosphate pathway (Pentose phosphate cycle) | 1.50E-04 |
| Amino sugar and nucleotide sugar metabolism | 1.54E-04 |
| Renal cell carcinoma | 1.78E-04 |
| Fc gamma R-mediated phagocytosis | 3.81E-04 |
| Phagosome | 4.05E-04 |
| Pentose phosphate pathway | 7.36E-04 |
| Mitophagy - animal | 1.02E-03 |
| Central carbon metabolism in cancer | 1.02E-03 |
| Lysosome | 3.24E-03 |
| Pentose phosphate pathway, oxidative phase, glucose 6P => ribulose 5P | 3.47E-03 |
| Collecting duct acid secretion | 3.97E-03 |
| Synaptic vesicle cycle | 4.33E-03 |
| Keratan sulfate degradation | 5.70E-03 |
| Insulin signaling pathway | 6.87E-03 |

Figure S1. The nonnormal distribution (A) and normal Q-Q plot (B) of *ACLY* expression values in our AML patients. Normal distribution test using Shapiro-Wilk method. P value <0.001.


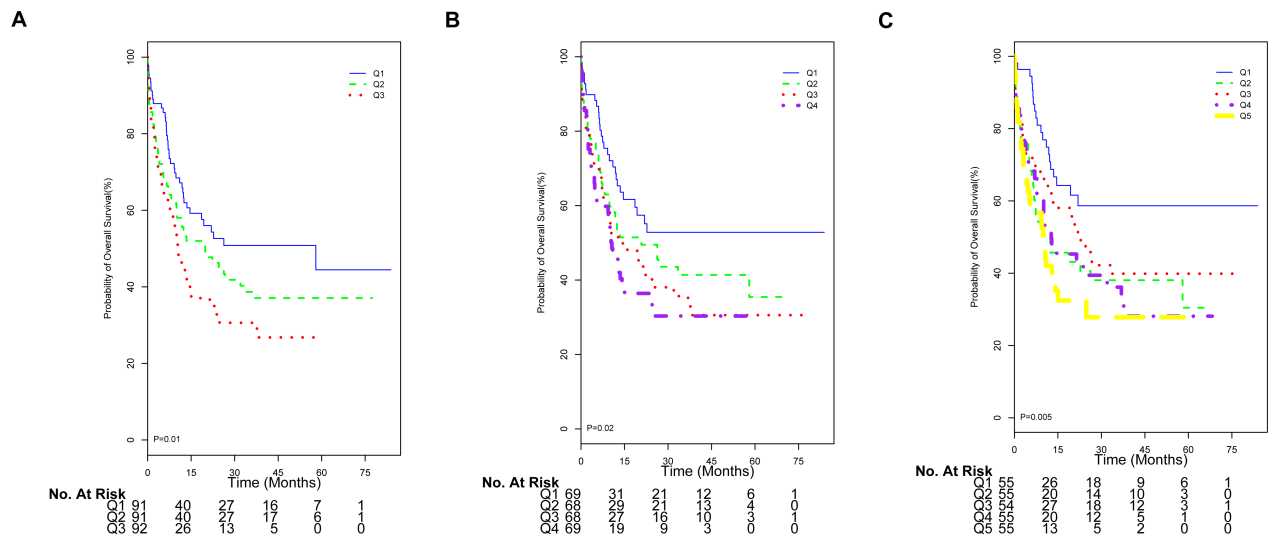


Figure S2. Survival curves of our AML patients. Kaplan-Meier estimates of OS by 3(A), 4(B) and 5(C) categorical expression of ACLY based on 3, 4 and 5 quartiles, respectively.


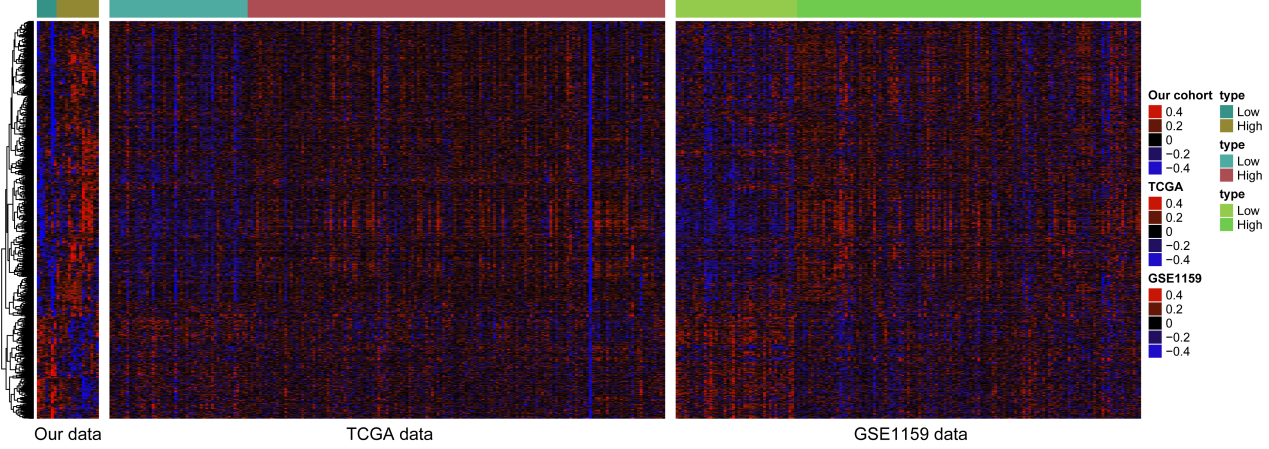


Figure S3. Heatmap plot illustrating the miRNAs expression between high and low ACLY expression in three different cohorts.


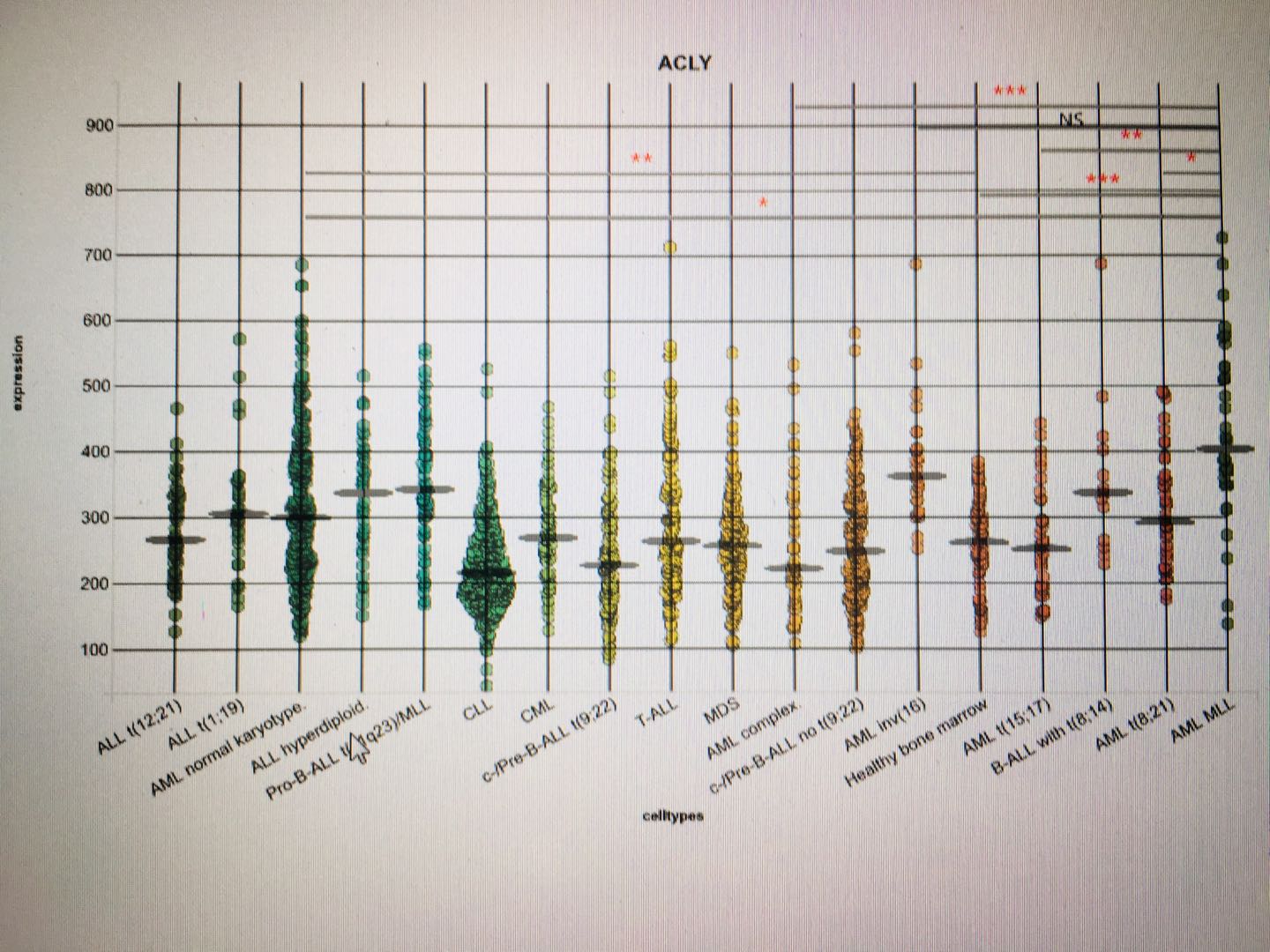


Figure S4. ACLY expression analyzed by Bloodspot. Human AML, ALL and preleukemic stages cells are from [GSE13159](http://www.ncbi.nlm.nih.gov/geo/query/acc.cgi?acc=GSE13159); ALL hyperdiploid; ALL with hyperdiploid karyotype, ALL t(12;21); ALL with t(12;21), ALL t(1;19); ALL with t(1;19), AML complex; AML complex aberrant karyotype, AML inv(16); AML with inv(16)/t(16;16), AML normal karyotype; AML with normal karyotype + other abnormalities, AML MLL; AML with t(11q23)/MLL, AML t(15;17); AML with t(15;17), AML t(8;21); AML with t(8;21),


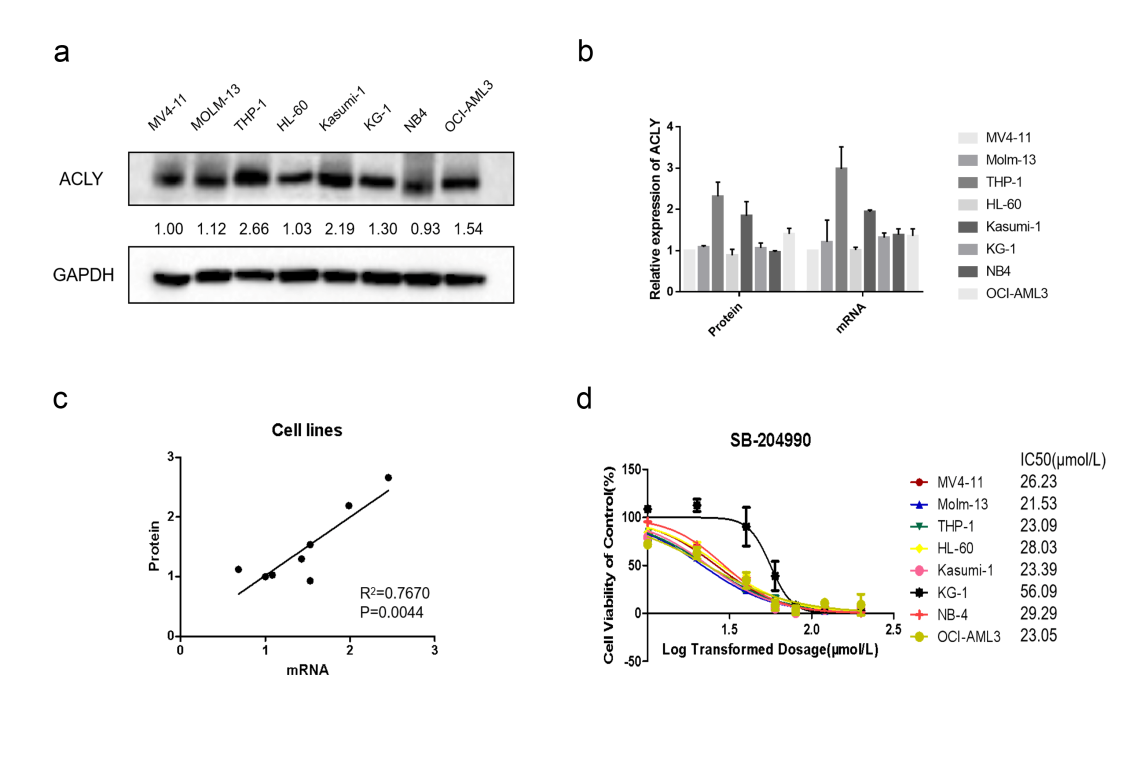


Figure S5. SB-204990 induces cell growth arrest in AML cell lines. ACLY protein(a,b) and mRNA(b) levels in AML cell lines. ACLY mRNA expression collerates with protein expression(c).AML cell lines were treated with SB-204990 96h at 0,10,20,40,60,80,120 umol/L(d). IC50 was calculated and shown in the figure.Values shown are mean ±SEM from 3 independent experiments. MV4-11(AML, FLT-ITD,MLL-AF4), MOLM-13(AML,FLT-ITD),THP-1(AML, MLL/AF9, NRAS G12D), HL-60(AML, NRAS Q61L),Kasumi-1(AML, AML1-ETO, KRAS G12D),KG-1(AML, TP53 mutation), NB4(APL, TP53 mutation),OCI-AML3(AML, DNMT3A R882C).


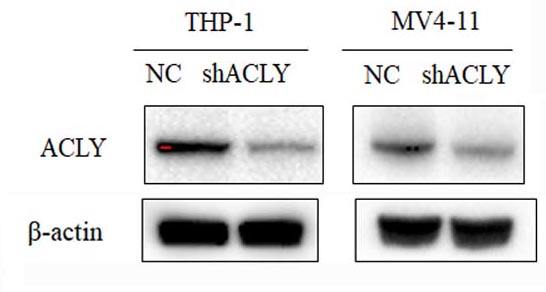


Figure S6. Knockdown ACLY in AML cell lines.
